# Mind-wandering and alterations to default mode network connectivity when listening to naturalistic versus artificial sounds

**DOI:** 10.1038/srep45273

**Published:** 2017-03-27

**Authors:** Cassandra D. Gould van Praag, Sarah N. Garfinkel, Oliver Sparasci, Alex Mees, Andrew O. Philippides, Mark Ware, Cristina Ottaviani, Hugo D. Critchley

**Affiliations:** 1 Department of Psychiatry, Brighton and Sussex Medical School, Brighton, UK; 2 Sackler Centre for Consciousness Science, University of Sussex, Brighton, UK; 3 Brighton and Sussex Medical School, Brighton, UK; 4 Department of Informatics, University of Sussex, Brighton, UK; 5 Wavelength Project, Exeter, UK; 6 Neuroimaging Laboratory, Santa Lucia Foundation, Rome, Italy

## Abstract

Naturalistic environments have been demonstrated to promote relaxation and wellbeing. We assess opposing theoretical accounts for these effects through investigation of autonomic arousal and alterations of activation and functional connectivity within the default mode network (DMN) of the brain while participants listened to sounds from artificial and natural environments. We found no evidence for increased DMN activity in the naturalistic compared to artificial or control condition, however, seed based functional connectivity showed a shift from anterior to posterior midline functional coupling in the naturalistic condition. These changes were accompanied by an increase in peak high frequency heart rate variability, indicating an increase in parasympathetic activity in the naturalistic condition in line with the Stress Recovery Theory of nature exposure. Changes in heart rate and the peak high frequency were correlated with baseline functional connectivity within the DMN and baseline parasympathetic tone respectively, highlighting the importance of individual neural and autonomic differences in the response to nature exposure. Our findings may help explain reported health benefits of exposure to natural environments, through identification of alterations to autonomic activity and functional coupling within the DMN when listening to naturalistic sounds.

Naturalistic sounds and ‘green’ environments are empirically reported to be subjectively more pleasant than artificial sounds and environments^1^,^2^,^3^. The positive effects of exposure to naturalistic environmental stimuli extend to health benefits, including improvements in the patient experience of general anaesthesia^4^, enhanced post-operative recovery^5^,^6^, and reduced pain and anxiety in hospice care^7^. These effects are observed following controlled exposure to naturalistic stimuli re-created in an experimental setting. A primary focus of research in this area relates to the ‘restorative’ effects of naturalistic stimuli, and assessing the ability of nature exposure to promote recovery from physiological or psychological stress. These restorative effects are framed in the context of two prevailing theories: 1) attentional restoration theory (ART)^8^ and; 2) stress recovery theory (SRT)^9^. ART proposes that an artificial environment is saturated with stimuli that impose a high level of cognitive and attentional demand. In contrast, stimuli derived from natural environments are proposed to engage less with top-down drivers of ‘directed attention’. Exposure to naturalistic stimuli might thus provide temporal respite from attentional load, thereby promoting recovery of attentional capacity. In contrast, SRT posits that physiological (autonomic) and psychological stress are reduced within naturalistic compared to artificial environmental contexts as a consequence of human evolutionary adaptation to naturalistic stimuli. SRT makes a more direct link between nature exposure and physiological shifts in autonomic balance toward parasympathetic (‘rest-digest’) activation, with a concomitant reduction in sympathetic (‘fight-flight’) activation within the cardiovascular system.

Increases in cognitive capacity (ART) are observed across specific domains following exposure to naturalistic stimuli^10^,^11^,^12^. These effects may be amplified in individuals experiencing high levels of self-reported cognitive exhaustion^11^. However, the cognitive benefits that are central to the ART model provide no proximate explanation for the physiological effects induced by naturalistic environments^13^. Psychological factors do, however, drive stress-related changes in bodily physiology. Techniques such as simulated interviews^14^ and backwards counting tasks^15^ are used in experimental studies of mental stress and associated with states of bodily arousal^14^. Exposure to naturalistic stimuli following psychological stress challenge can increase parasympathetic activation^14^ and reduce sympathetic activation^15^, as indexed by electrodermal activity, heart rate and blood pressure, or cortisol levels. These physiological changes are not always accompanied by changes in subjective ratings of anxiety^14^.

Brain imaging studies can help determine the neurobiological mechanisms underlying these behavioural and physiological observations. However, as yet there have been relatively few imaging investigations into the restorative effects of nature exposure. A field study using electroencephalography identified an increase in δ band power when participants transitioned from urban to natural environments^16^. This was interpreted as demonstrating a reduction in neural correlates of arousal and frustration, and an increase in active engagement in the naturalistic environment, thereby supporting the ART model. In a functional magnetic resonance imaging (fMRI) study, participants were presented with images of beaches (naturalistic ‘tranquil’ condition) or freeways (artificial ‘non-tranquil’ condition) while they listened to a soundtrack which could be interpreted as both rolling waves or high speed traffic^17^. During the tranquil condition greater functional neural coupling was observed between auditory cortex and medial prefrontal cortex, posterior cingulate, temporo-parietal cortex and thalamus. The authors link the low attentional demands of the naturalistic stimuli with increased ‘default mode network’ (DMN) activity. The DMN describes a set of regions where activity is increased during ‘task free’ states of wakefulness, and is decreased during task performance associated with external cognitive load. In line with ART, increased activation of posterior cingulate cortex (PCC, a key DMN ‘hub’) was observed during the tranquil condition relative to a baseline no-stimulus condition, yet surprisingly there were no supra-threshold differences when contrasting tranquil and non-tranquil stimulation. A limitation in this study, however, is the lack of behavioural or physiological data to support the interpretation of neural findings.

ART and SRT make clear predictions about brain activity in task free or ‘mind-wandering’ situations. If exposure to naturalistic stimuli reduces cognitive load relative to artificial stimuli, as suggested by ART, one might predict an increase in mind wandering and DMN activity in naturalistic versus artificial conditions, consistent with an increase in task-free activity^17^. Alternately, if exposure to naturalistic versus artificial stimuli results in more general stress reduction associated with alterations in autonomic activation, one might observe DMN differences that mirror changes in parasympathetic-sympathetic balance. In the present fMRI study, we measured changes in the activation and connectivity of the DMN during exposure to naturalistic and artificial stimuli, along with a no-soundscape control condition, to investigate and test the opposing ART and SRT hypotheses. We used whole brain seeded timecourse correlations with the PCC to investigate DMN functional connectivity under each condition. We used the functional connectivity map of the DMN under the control condition to test the ART hypothesis of increased activation in DMN areas during naturalistic conditions, and assessed alterations in connectivity through comparison of connectivity maps between conditions. We also conducted an exploratory analysis of anterior and posterior salience networks, the dorsal attention network, and the executive control network^18^, to test whether alterations to connectivity within these systems can account for the increased cognitive capacity observed following exposure to naturalistic stimuli (ART).

Participants were exposed to conditions of artificial and naturalistic ‘soundscapes’, comprised of equally weighted familiar and unfamiliar sounds, which were rated for pleasantness, intensity and familiarity using a visual analogue scale (VAS). The fMRI data acquisition was accompanied by behavioural measures of attentional deployment using reaction times in performance of a low cognitive load tracking task, and subjective indices of attentional capture in the form of VAS measures of task engagement, rumination, distraction by thoughts and distraction by the soundscapes themselves. Neural, behavioural and subjective data were complemented by physiological measurement of arousal indexed by changes in heart rate and heart rate variability (HRV). HRV analysis involves spectral and temporal decomposition of the intervals between successive heart beats. Interpretation of low frequency (0.04–0.15 Hz) components of HRV is complicated by contributions from both sympathetic and parasympathetic responses^19^; we therefore constrained our HRV analysis to assessment of the high frequency (0.15–0.4 Hz) component, as a reliable index of cardiac parasympathetic activity^20^,^21^,^22^,^23^,^24^. We hypothesised that reaction times would be increased in the artificial condition compared to the naturalistic, suggesting a relative disengagement of attention from the task (e.g. through distraction or mind-wandering), but that the neural and physiological data would support SRT in providing evidence that exposure to naturalistic stimuli would enhance cardiac parasympathetic activity (mediating reported health benefits associated with nature exposure), underpinned by changes in functional neural connectivity which support the differences in phenomenological experience of naturalistic and artificial exposure conditions.

## Results

### Attentional monitoring

**Figure 1:**
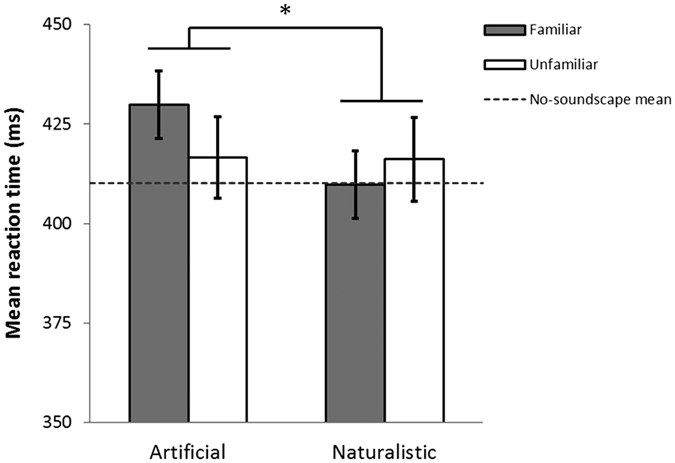
Mean reaction times in attentional monitoring task whilst listening to familiar and unfamiliar, artificial and naturalistic soundscapes. After controlling for variance in reaction times in the no-soundscape condition (dashed line), the main effect of artificiality indicates increased reaction times in artificial compared to naturalistic conditions (*p* = 0.029). *p < 0.05. Error bars ± 1 SEM.

The exposure conditions consisted of: (1) artificial familiar; (2) artificial unfamiliar; (3) naturalistic familiar; (4) naturalistic unfamiliar; (5) no-soundscape (control). Each soundscape lasted 5 minutes 25 seconds, and was presented in a randomised order while fMRI data were acquired and attention was monitored using a low level reaction time task. Reaction times in the attentional monitoring task were significantly increased in the artificial condition (*μ* = 423.22 ms) compared to naturalistic condition (*μ* = 412.98 ms) (main effect of artificiality: *F*_(1,14)_ = 5.94, *p* = 0.029) (Fig. 1). There was no main effect of familiarity (*F*_(1,14)_ = 1.31, *p* = 0.272), and no artificiality-by-familiarity interaction (*F*_(1,14)_ = 0.25, *p* = 0.623). Post-hoc t-tests demonstrated a significant difference between reaction times in the artificial familiar condition (*μ* = 432.98 ms) compared to the naturalistic familiar condition (*μ* = 415.04 ms) (*t*_(16)_ = 3.07, *p* = 0.007). To minimise the number of exploratory comparisons, the artificial familiar and naturalistic familiar conditions were taken forward for further analysis of subjective, physiological and neural effects.

### Subjective ratings and physiological effects

**Figure 2:**
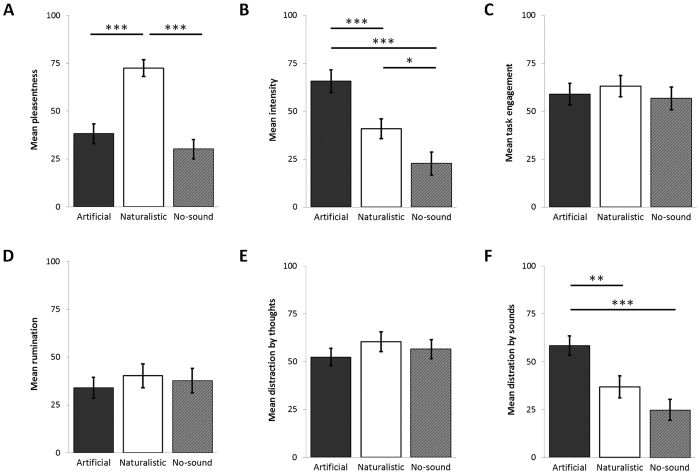
Subjective ratings of (**A**) pleasantness, (**B**) intensity of sounds, (**C**) task engagement, (**D**) rumination, (**E**) distraction by thoughts, and (**F**) distraction by sounds, for artificial familiar, naturalistic familiar and no-soundscape conditions. Error bars ± 1SEM. *p < 0.05; **p < 0.01; ***p < 0.001.

At the end of each condition, participants completed a set of visual analogue scales to rate subjective experience. There was a significant effect of soundscape on pleasantness (*F*_(2,32)_ = 35.57, *p* < 0.001) (Fig. 2A) and intensity of the sounds (*F*_(2,32)_ = 16.41, *p* < 0.001) (Fig. 2B), with the naturalistic familiar soundscape rated as more pleasant (*μ* = 74.30) than both the artificial familiar (*μ* = 38.19) (*t*_(16)_ = 6.57, *p* < 0.001) and the no-soundscape control condition (*μ* = 28.94) (*t*_(16)_ = 6.34, *p* < 0.001), and less intense (*μ* = 41.86) than the artificial familiar (*μ* = 65.73) (*t*_(16)_ = 4.42, *p* < 0.001) and the no-soundscape condition (*μ* = 24.04) (*t*_(16)_ = 5.18, *p* < 0.001). There was no effect of soundscape on the subjective rating of perceived task engagement (*F*_(2,32)_ = 0.42, *p* = 0.663) (Fig. 2C). In relation to thought content, there was no effect of soundscape on rumination (*F*_(2,32)_ = 0.90, *p* = 0.420) (Fig. 2D), or distraction by thoughts (*F*_(2,32)_ = 1.24, *p* = 0.304) (Fig. 2E). There was a significant effect of soundscape on distraction by sounds (*F*_(2,32)_ = 15.54, *p* < 0.001) (Fig. 2F) with the artificial familiar sounds (*μ* = 58.36) being significantly more distracting than the naturalistic familiar sounds (*μ* = 36.82) (*t*_(16)_ = 3.27, *p* = 0.005), and the no-soundscape control condition (*μ* = 24.77) (*t*_(16)_ = 5.62, *p* < 0.001). There was no difference in the distraction by sounds between the naturalistic familiar condition and the no-soundscape condition (*t*_(16)_ = 2.11, *p* = 0.051).

**Figure 3:**
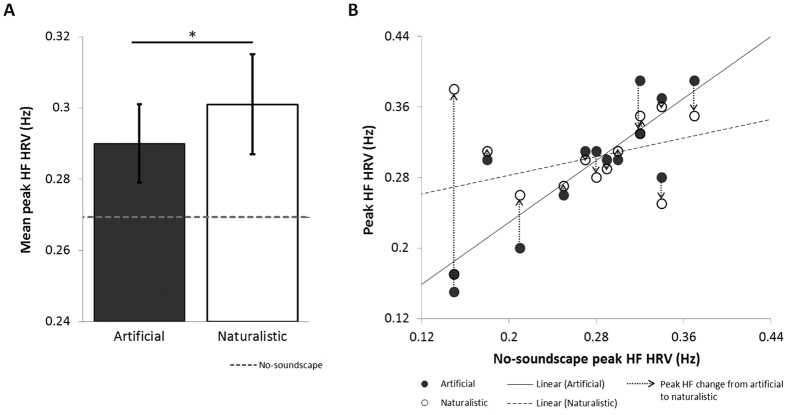
(**A**) Significant increase in the mean peak of the high frequency component of heart rate variability (peak HF HRV) in naturalistic familiar compared to artificial familiar conditions, after controlling for the baseline (no-soundscape) high frequency peak. Error bars ± 1 SEM. *p < 0.05 (**B**) Interaction between baseline (no-soundscape) peak HF and artificial familiar (dark circle, solid line) and naturalistic familiar (white circle, dashed line) peak HF. Individuals with low baseline peak HF show an increase in parasympathetic tone from artificial to naturalistic conditions (upwards arrow); individuals with high baseline peak HF show a decrease in parasympathetic tone from artificial to naturalistic conditions (downwards arrow).

During the experiment cardiac activity was continuously monitored using pulse oximetry. There was no main effect of soundscape on heart rate (*F*_(2,28)_ = 3.06, *p* = 0.063), suggesting there were no significant differences in broad arousal between the artificial familiar (*μ* = 65.75 bpm), naturalistic familiar (*μ* = 67.31 bpm) and no-soundscape condition (*μ* = 68.24 bpm). However, after controlling for baseline heart rate variability, there was a significant increase in the peak frequency of the high frequency band (peak HF) in the naturalistic familiar compared to artificial familiar condition (*F*_(1,12)_ = 8.58, *p* = 0.013), suggesting an increase in parasympathetic activity in the naturalistic compared to artificial condition (Fig. 3A). The large variance in peak HF reflected the significant interaction between baseline peak HF and stimulus condition (*F*_(1,12)_ = 8.07, *p* = 0.015), where individuals with low baseline peak HF experienced an increase in parasympathetic activity in naturalistic compared to artificial conditions, while individuals with high baseline peak HF experienced a decrease in parasympathetic activity in naturalistic compared to artificial conditions (Fig. 3B). There were no significant differences in absolute (*F*_(1,12)_ = 0.19, *p* = 0.671) or relative (percentage) high frequency power (*F*_(1,12)_ = 0.02, *p* = 0.897).

### Functional brain imaging findings

**Figure 4:**
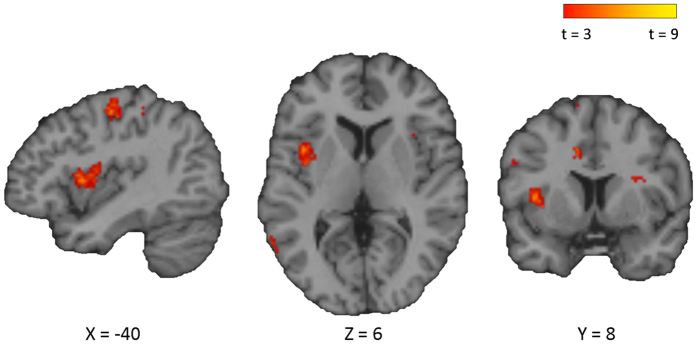
Region of significantly increased activation in the naturalistic familiar condition compared to the artificial familiar condition in the middle insula of the left hemisphere (*p*_(*FWE clus.*)_ 0.002, *k* = 96, *p*_(*FWE peak*)_ = 0.979, *Z* = 4.09, [−40, 8, 6]). Note precentral cluster in the sagittal view does not survive FWE correction (*p*_(*FWE clus.*)_ = 0.050, *k* = 55, *p*_(*FWE peak*)_ = 0.986, *Z* = 4.05, [−36, −14, 62]).

Neural data were acquired using fMRI during each of the experimental and control conditions. For general linear model analysis of evoked changes in neural activity within the DMN, a canonical DMN mask was generated using seed based functional connectivity with the PCC during the no-soundscape control condition. There were no suprathreshold differences of activation within the DMN masked area between the artificial familiar and naturalistic familiar conditions (*p*_(*FWE peak*)_ > 0.05, *p*_(*FWE clus.*)_ > 0.05). There was, however, a region of significantly increased activation during in the naturalistic familiar condition compared to the artificial familiar condition outside of DMN areas, in the middle insula of the left hemisphere (*p*_(*FWE clus.*)_ = 0.002, *k* = 96, *p*_(*FWE peak*)_ = 0.979, *Z* = 4.09, [40, 8, 6]) (Fig. 4).

**Figure 5:**
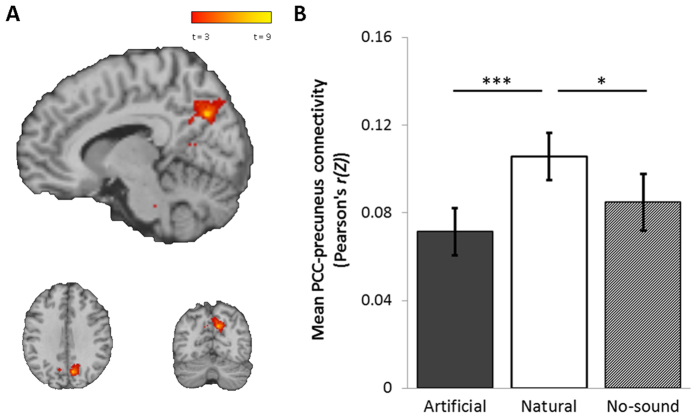
(**A**) FWE corrected significant region of increased local functional connectivity between the PCC seed region and the precuneus in the naturalistic familiar condition compared to artificial familiar condition (*p*_(*FWE clus*)_ < 0.001, *k* = 183, *p*_(*FWE peak*)_ = 0.058, *Z* = 4.98, [10, −68, 34]). (**B**) Main effect of soundscape demonstrated in the extracted connectivity scores between the PCC and precuneus (*F*_(2,32)_ = 10.96, *p* = 0.001), with increased connectivity in the naturalistic familiar (*μ* Pearson’s *r(Z*) = 0.11) compared to the artificial familiar (*μ* Pearson’s *r(Z*) = 0.07) (*t*_(16)_ = 6.35, *p* < 0.001) and no-soundscape condition (*μ* Pearson’s *r(Z*) = 0.85) (*t*_(16)_ = 2.94, *p* = 0.010). Error bars ± 1 SEM. *p < 0.05 **p < 0.01 ***p < 0.001.

**Figure 6:**
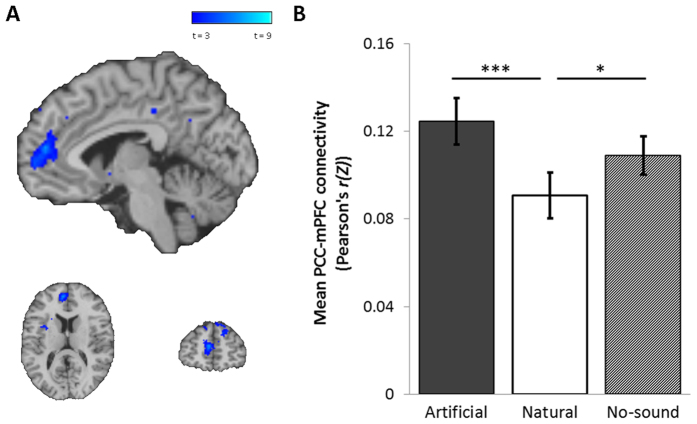
(**A**) FWE corrected significant region of reduced functional connectivity between the PCC seed and the mPFC in the naturalistic familiar condition compared to the artificial familiar condition (*p*_(*FWE clus.*)_ < 0.001, *k* = 185, *p*_(*FWE peak*)_ = 0.770, *Z* = 4.29, [−4, 52, 14]). (**B**) Main effect of soundscape on extracted connectivity scores between the PCC and mPFC (*F*_(2,32)_ = 9.94, *p* < 0.001), with decreased connectivity in the naturalistic familiar condition (*μ* Pearson’s *r(Z*) = 0.91) compared to the artificial familiar (*μ* Pearson’s *r(Z*) = 0.13) (*t*_(16)_ = 4.90, *p* < 0.001) and no-soundscape condition (*μ* Pearson’s *r(Z*) = 0.11) (*t*_(16)_ = 2.35, *p* = 0.032). Error bars ± 1 SEM. *p < 0.05 **p < 0.01 ***p < 0.001.

In functional connectivity analysis, the DMN was identified separately for each condition by the extraction of timecourse activation data from the PCC, and entered into regression analysis against all voxels to identify significant correlations between the PCC and all other regions of the brain. In contrasting the DMN functional connectivity maps, the naturalistic familiar condition was associated with a significant increase in connectivity between PCC and the precuneus (*p*_(*FWE clus.*)_ < 0.001, *k* = 183, *p*_(*FWE peak*)_ = 0.058, *Z* = 4.98, [10, −68, 34]) (Fig. 5), and a decrease in connectivity between the PCC and the medial prefrontal cortex (mPFC) (*p*_(*FWE clus.*)_ < 0.001, *k* = 185, *p*_(*FWE peak*)_ = 0.770, *Z* = 4.29, [−4, 52, 14]) (Fig. 6), when compared to the artificial or no-sound control conditions.

**Figure 7:**
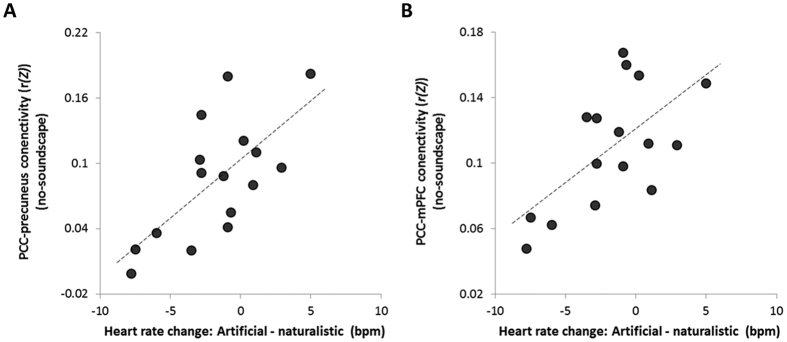
Correlation between changes in arousal (change in heart rate between the artificial familiar and naturalistic familiar condition) and functional connectivity scores in the baseline no-soundscape condition between the PCC seed and (**A**) the precuneus (Pearson’s *r* = 0.68, *p* = 0.003) and (**B**) the mPFC (Pearson’s *r* = 0.628, *p* = 0.009).

Finally, relationships were explored between the change in arousal (artificial heart rate – naturalistic heart rate) and functional connectivity. There was a significant positive correlation between the change in arousal and baseline (no-soundscape) functional connectivity between the PCC and the precuneus (Pearson’s *r* = 0.68, *p* = 0.003) (Fig. 7A), and between the change in arousal and baseline PCC and mPFC connectivity (Pearson’s *r* = 0.63, *p* = 0.009) (Fig. 7B).

**Figure 8:**
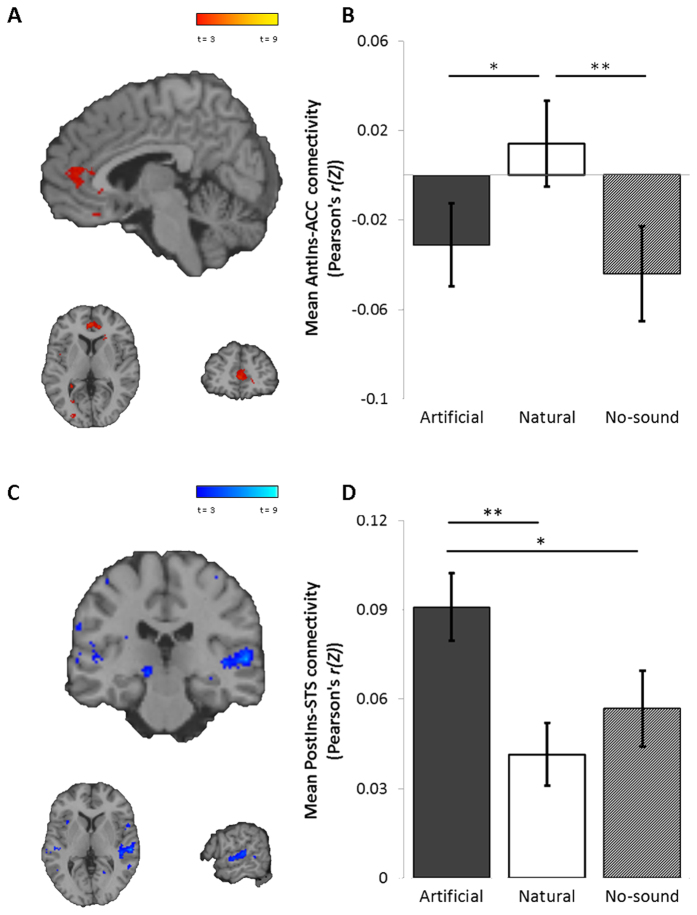
Exploratory connectivity analysis of salience networks, seeded from (**A**,**B**) bilateral anterior insula; and (**C**,**D**) bilateral posterior insula. (**A**) The anterior insula show increased functional connectivity with the ACC (*p*_(*FWE clus.*)_ = 0.021, *k* = 84, *p*_(*FWE peak*)_ = 0.995, *Z* = 3.88, [2, 46, 4]) in the naturalistic compared to artificial condition. (**B**) Main effect of soundscape on extracted connectivity scores between the anterior insula and ACC, with increased functional connectivity in the naturalistic condition compared to the artificial (*t*_(16)_ = 2.71, *p* = 0.016) and no-soundscape control condition (*t*_(16)_ = 3.09, *p* = 0.007). (**C**) The posterior insula show increased functional connectivity with a region of the right STS (*p*_(*FWE clus.*)_ = 0.002, *k* = 121, *p*_(*FWE peak*)_ = 0.511, *Z* = 4.54, [60, −24, 4]) in the artificial condition compared to the naturalistic condition. (**D**) Main effect of soundscape on extracted connectivity scores between the posterior insula and the right STS, with increased functional connectivity in the artificial condition compared to the naturalistic (*t*_(16)_ = 5.01, *p* < 0.001) and no-soundscape control condition (*t*_(16)_ = 2.85, *p* = 0.012). Error bars ± 1 SEM. *p < 0.05 **p < 0.01 ***p < 0.001.

In exploratory analysis of functional connectivity in the salience (bilateral anterior and posterior insula seeds), dorsal attention (right supramarginal gyrus seed)and executive control networks (left and right dorsolateral prefrontal cortex seeds), we found differential effects of naturalistic and artificial soundscapes in the salience networks only (see Fig. 8). All other differences were non-significant after FWE correction at the cluster or peak level. In the anterior salience network there was a significant increase in connectivity in the naturalistic familiar condition compared to the artificial condition, between bilateral anterior insula and the anterior cingulate cortex (ACC) (*p*_(*FWE clus.*)_ = 0.021, *k* = 84, *p*_(*FWE peak*)_ = 0.995, *Z* = 3.88, [2, 46, 4]) (Fig. 8A). In the posterior salience network there was a significant increase in connectivity in the artificial familiar condition compared to the naturalistic condition, between bilateral posterior insula and a region of the right superior temporal sulcus (STS) (*p*_(*FWE clus.*)_ = 0.002, *k* = 121, *p*_(*FWE peak*)_ = 0.511, *Z* = 4.54, [60, −24, 4]) (Fig. 8B). Analysis of the extracted connectivity strength for the anterior salience network from each condition shows the activation timecourse in the anterior insula to be positively correlated with the timecourse of the anterior cingulate in the naturalistic familiar condition (*μ* Pearson’s *r(Z*) = 0.01), while activity in the anterior insula is negatively correlated with the anterior cingulate in the artificial (*μ* Pearson’s *r(Z*) = −0.03) and no-soundscape control conditions (*μ* Pearson’s *r(Z*) = −0.04). There was a significant increase in connectivity between the anterior insula and the anterior cingulate in the naturalistic condition compared to the artificial (*t*_(16)_ = 2.71, *p* = 0.016) and no-soundscape control condition (*t*_(16)_ = 3.09, *p* = 0.007). In the posterior salience network, there was a significant increase in positive connectivity between the posterior insula and the superior temporal sulcus in the artificial condition (*μ* Pearson’s *r(Z*) = 0.09) compared to the naturalistic (*μ* Pearson’s *r(Z*) = 0.04, *t*_(16)_ = 5.01, *p* = <0.001) and no-soundscape control condition (*μ* Pearson’s *r(Z*) = 0.06, *t*_(16)_ = 2.85, *p* = 0.012). There were no significant correlations between baseline connectivity scores and the change in heart rate or HRV measures after controlling for multiple comparisons (*p* > 0.05).

## Discussion

We tested from a neurobiological perspective two competing theoretical accounts of the reported restorative effects of exposure to naturalistic environmental stimuli. We assessed activation and functional connectivity within the DMN when participants were listening to naturalistic familiar and artificial familiar soundscapes, encompassing regions whose activity reduces with externally directed attention and cognitive load. We further explored changes in connectivity within the salience, dorsal attention and executive control networks. Behavioural results showed that artificial soundscapes were associated with poorer attentional monitoring compared to naturalistic soundscapes (Fig. 1). Subjective reports indicated that the largest differences between artificial familiar and naturalistic familiar conditions were observed in ratings of pleasantness (Fig. 2A), intensity (Fig. 2B), and distraction by the sounds themselves (Fig. 2F). We also observed a significant increase in the high frequency peak of HRV during the naturalistic familiar compared to artificial familiar condition (Fig. 3A), indicating an increase in cardiac parasympathetic activity. Importantly, the shift in high frequency peak between conditions was dependant on the baseline peak level: individuals with low baseline parasympathetic activity showed an increase in parasympathetic activity in the naturalistic condition. In contrast, individuals with high baseline parasympathetic activity showed a relative decrease in parasympathetic activity in the naturalistic condition (Fig. 3B). We observed no significant difference in the absolute and relative power of high frequency HRV, however this effect may have been masked by difference in respiratory activity^20^.

The behavioural and subjective data show partial support for ART, by demonstrating a reduced capacity for external attentional monitoring and an increase in attentional capture by artificial soundscapes. The increase attentional capture of the artificial soundscapes compared to naturalistic or control is also demonstrated in exploratory analysis of the salience networks, which show increased connectivity with auditory cortex in the artificial condition (Fig. 8B), but increased connectivity with limbic structures in the naturalistic condition (Fig. 8A). However, our findings provide no evidence to support the ART-related hypothesis of an increase in DMN (task free) activity in the naturalistic familiar condition compared to the artificial familiar condition, or an increase in the subjective experience of rumination or mind wandering. Rather, the regional localisation of alterations in functional connectivity of the DMN appears to reflect a shift in autonomic balance in line with SRT, where we observed increased coupling between the PCC and precuneus in the naturalistic familiar condition (Fig. 5) and decreased coupling between the PCC and mPFC (Fig. 6). These differences were significant both in comparison to the artificial familiar condition, and when comparing the naturalistic familiar with no-soundscape condition, suggesting these effects are induced specifically by naturalistic familiar soundscapes. We note that the differences in functional connectivity are strong even after controlling for differences in subjective ratings, suggesting that the neural effects are not due perceived pleasantness, intensity or distraction by soundscapes.

Activity and connectivity of the DMN is coupled to changes in autonomic activation^25^,^26^,^27^. A recent meta-analysis of fMRI investigations assessing the patterns of brain activity related to autonomic responses suggested that sympathetic activation was associated with an increase in neural response in areas associated with executive function and salience, while parasympathetic activation involved areas of the DMN^25^. Tasks which result in sympathetic activation evoked activity changes within regions including the ventromedial prefrontal cortex and pregenual anterior cingulate, while tasks resulting in parasympathetic activation identified clusters in the precuneus and dorsal PCC^25^. Our interpretation that differential anterior and posterior changes in DMN functional connectivity are directly coupled to physiology, notably specific changes in parasympathetic cardiac drive during the naturalistic familiar condition, is supported by this meta-analysis. Our findings also contribute to growing empirical description of brain mediators of stress-related baroreflex suppression (hence sympathovagal balance)^28^. However, ventromedial prefrontal cortex (a component of the DMN) appears strongly antisympathetic^29^,^30^,^31^ a role that does not come through in this meta-analysis. Our neural data suggest that an increased capacity for external attentional monitoring in the naturalistic condition is associated with the overall increase in parasympathetic activity compared to artificial familiar conditions. This inference is also supported by the observed increase in peak high frequency HRV in the naturalistic familiar condition. We note that the physiological arousal effects are correlated with individual differences in baseline functional connectivity between the DMN hubs (Fig. 7) and baseline parasympathetic activity (Fig. 3B). These individual differences suggest that arousal may be related to basal state of neural connectivity and the participant’s current autonomic state. This demonstration of individual differences in physiological and neural response to naturalistic stimuli may be in part responsible for inconsistences with regard to arousal effects in previous investigations, and emphasise a need for further investigation.

Our focus on the DMN was motivated by a desire to understand the task-free effects of naturalistic stimulus exposure. Alterations in DMN functional connectivity are reported in association with disorders related to psychological stress, including anxiety^32^, post-traumatic stress disorder^33^ and depression^34^. Dysfunctional regulation of the DMN is linked to intrinsic alterations in functional connectivity within the network itself 35 and dysregulation in the competition between the DMN and anti-correlated task-positive networks. Anxiety disorders are typically associated with an increase in functional connectivity or neural activity in the mPFC and ACC^33^,^34^, a region which is associated with metalizing^36^, evaluative and self-referential processing^37^,^38^ and sympathetic cardiovascular drive^39^,^40^. These accounts suggest that increased connectivity with mPFC and ACC subregions is associated with an increase in self-referential thought processes. Our findings of decreased functional connectivity between the PCC and mPFC hubs of the DMN in naturalistic familiar conditions may therefore correspond to an attenuation of self-referential thought processes during exposure to naturalistic environmental stimuli. However, the increased functional connectivity between the anterior insula and ACC identified in our exploratory analysis may suggest an increase in emotional salience under natural conditions^41^. Conversely, the precuneus and PCC are thought to support broad monitoring of external and internal self-generated experience^42^, including visuo-spatial imagery^43^ and working memory^44^, with the precueneus specifically linked to relaxed states of consciousness which involve higher order self-representation, as opposed to states of active task engagement^43^,^45^. Our observation of increased functional connectivity between the PCC and precuneus regions of the DMN during the naturalistic familiar condition may suggest an increase in broad integrative monitoring and visual working memory during naturalistic exposure.

This interpretation of differential connectivity of the anterior and posterior midline DMN structures is in accord with the more general notion that the medial prefrontal cortex component of the DMN is associated with inward-directed focus, in contrast to the precuneus/PCC which is association with outward-directed focus of attentional processing^46^,^47^. We found no statistical difference in the ratings of rumination or distraction by internal thoughts to suggest a qualitative difference in the ‘direction of thought focus’ between the conditions, however, this may be due to the relatively brief stimulation period (5 min 25 second) for each condition. It is possible that the short duration and switching between soundscapes was sufficiently stimulating that mind wandering and internal thought processes were maintained at low levels throughout. Future investigations of these effects may benefit from employing longer exposure durations, to increase the likelihood of mind wandering. Extended exposure durations may also increase the likelihood of detecting differences in the degree of DMN activation between artificial and naturalistic conditions, alongside the more subtle differences in connectivity reported here.

ART proposes that naturalistic environments are restorative through the provision of respite from directed attentional demands, which is anticipated to engage an increase in DMN (or task-free) activity^17^. Although we identified an increase in attentional capacity during naturalistic familiar compared to artificial familiar conditions, we found no evidence within the neural data for an increase in task-free activity during this relatively short exposure to naturalistic familiar conditions. The primary claims of ART, however, relate to post-exposure, rather than peri-exposure effects; a limitation in the present study may therefore be the focus on neural, physiological and psychological alterations identified during naturalistic/artificial exposure rather than after the exposure session. We also note that the differences in attentional monitoring capacity were only significant for familiar and not unfamiliar conditions (Fig. 1). This may suggest that the attentional demand of artificial conditions is negated by the uncertainty of a naturalistic environment comprised of unfamiliar stimuli.

The myriad of purported health benefits ascribed to exposure to naturalistic stimuli may have a physiological homologue of ‘comfort’ in terms of a shift from sympathetic toward parasympathetic activation. Psychological or physiological stress is associated with heightened sympathetic activation and a withdrawal of peripheral parasympathetic tone. If the stress is chronic, this state of sustained autonomic imbalance is detrimental to health, and is recognised to contribute to cardiovascular disease and cellular aging^48^, obesity^49^, gastrointestinal disorders^50^ and a spectrum of mental health conditions, particularly depression^51^ and anxiety disorders. According to SRT, naturalistic environments tend to evoke increased parasympathetic tone as humans are evolutionarily adapted to natural environments. Thus SRT may provide a comprehensive account of beneficial psychophysiological effects of nature exposure following heightened states of arousal. Familiarity is likely to be an important mediator of the evoked psychophysiological comfort associated with nature, indeed our strongest behavioural effects of attentional monitoring were observed when contrasting the naturalistic and artificial familiar conditions only, and not the unfamiliar conditions.

In conclusion, we demonstrate that exposure to naturalistic familiar stimuli is associated with an increase in parasympathetic tone and alterations in DMN which reflect a shift in the autonomic balance towards parasympathetic activation in the naturalistic familiar condition and sympathetic activation in the artificial familiar condition, in accord with SRT. Individual differences in the neural and physiological response to naturalistic and artificial stimuli were associated with baseline autonomic state and baseline neural coupling. Alterations in autonomic balance are associated with a wide range of health effects, suggesting that SRT may provide a more complete account of the health benefits of nature exposure than ART. These data expand our current understanding of the restorative effects of nature by demonstrating differences in functional coupling between regions within the DMN, and suggest that environment plays a significant role in modulating our physiological, neural and psychological activity. Future investigations of DMN activity will be required to probe the specific shifts in thought patterns and content associated with changes in anterior and posterior midline coupling, and relate these more precisely to alterations in sympathetic/parasympathetic balance.

## Method

### Participants

All participants reported no history of significant medical, neurological or psychiatric illness and no long term medication usage. Ethical approval for conduct of the study was provided by Brighton and Sussex Medical School. All participants provided informed consent. All aspects of the investigation were performed in accordance with the Declaration of Helsinki.

17 healthy volunteers (mean age 26 years, range 21–34 years; seven female; two left handed) participated in fMRI data collection, during which time soundscapes were played, physiological data were recorded and participants undertook a low level attentional monitoring task.

### Soundscapes

The four distinct soundscapes of 5 minutes 25 second duration were comprised of seven individual sound clips. All sound clips were recorded using a professional quality Zoom H4n digital sound recorder (Zoom North America, New York, USA) with two Rode NTG1 Condenser Shotgun Microphones (RØDE Microphones, Sydney, Australia). 100 original 15 second sound clips were recorded and equalised for peak volume levels. Each clip was then rated for complexity (number of distinct sounds during each clip), consistency (number of significant changes in volume during a clip), familiarity and subjective intensity using a visual analogue scale (VAS). The 10 clips which scored the highest and lowest in familiarity were shortlisted for the familiar and unfamiliar, naturalistic and artificial conditions. From each shortlist, seven clips were selected for the main soundscape with intensity, complexity and consistency scores within the range of the mean ± two standard deviations of the combined shortlist average. These seven clips were looped and integrated to form the final composition for each condition.

### Attentional monitoring

Attentional capacity was assessed using a mind wandering task^52^ where participants monitored an unfilled white circle as it traversed the horizontal length of the stimulus display screen. At random intervals, the circle contour colour changed from white to red for 470 ms, then returned to white. Participants were instructed to press a button when they detected the colour change. Reaction times were calculated as the interval between the initiation of the colour change and the button press response. For each condition, the circle completed nine horizontal transits of the display area and changed colour 14 times (the total task duration was equal to the soundscape duration). Each participant completed five runs of the task with the accompanying soundscape played throughout the task through MRI compatible in-ear headphones (Etymotic Research Inc., Illinois, USA) or no soundscape for the control condition, assigned in a randomised order. All reaction time data was normally distributed (Kolmogorov–Smirnov *p* > 0.05). Differences in reaction times for each condition were assessed by a 2 × 2 ANCOVA with the factors ‘artificiality’ (artificial, naturalistic) and ‘familiarity’ (familiar, unfamiliar), and mean reaction time of the no-soundscape condition as a covariate. The artificial and naturalistic stimuli with the greatest difference in reaction time were taken forward for subjective, physiological and fMRI analysis.

### Subjective ratings and physiological recording

At the end of each run of fMRI data collection, participants provided feedback on their subjective experience during the attentional monitoring task and associated soundscape. Participants rated the pleasantness and intensity of the soundscape, their level of distraction attributed to the soundscape and their thoughts, their level of rumination and the degree to which they felt focused on the attentional task, each on a separate VAS.

Cardiac activity was recorded via pulse oximetry (8600FO Nonin Medical Inc., Minnesota, USA) during each run of the attentional monitoring task and used to determine heart rate (beats per minute) for each soundscape condition. Heart rate data was not available for two participants due to weak pulse oximetry signal.

#### Statistical analysis

All subjective and physiological data were assessed for normality using the Kolmogorov–Smirnov test and found to be not significantly different from a normal distribution (*p* > 0.05 in all cases). Subjective ratings were separately analysed using three-way ANOVAs, to assess differences between the no-soundscape control condition and the artificial and naturalistic soundscape conditions selected for further analysis. Change in subjective ratings and heart rate were calculated as the difference between artificial and naturalistic conditions (naturalistic - artificial). HRV analysis was performed using the HRVAS toolbox^53^, using the Lomb-Scargle periodogram method^54^,^55^ due to its suitability in handling irregular sampling of beat intervals^56^,^57^. HRV values were entered into a two-way ANCOVA, with the no-soundscape value included as a covariate. One participant was removed from HRV analysis as an outlier in the naturalistic familiar condition. Appropriate corrections were performed where the assumption of sphericity was violated. All statistical analyses were two-tailed with α set to *p* < 0.05.

### Magnetic resonance imaging

Neuroimaging data were acquired on a 1.5 T Siemens Avanto with 32 channel headcoil. Functional data consisted of T2*-weighted echo planar images (EPI) sensitive to Blood Oxygenation Level Dependent (BOLD) contrast (32 slices, 3 × 3 × 3 mm resolution, 20% inter-slice gap, TR = 2520 ms, TE = 43 ms), with 170 whole brain volumes acquired per run (total scanning time 35 minutes). The contrast contained within the average motion-correct EPI dataset from each participant was sufficient to infer corresponding structural anatomy at the same 3 mm spatial resolution.

#### Preprocessing

Data were preprocessed using SPM12 (Wellcome Trust Centre for Neuroimaging, University College London, UK) and in-house software implemented in MATLAB (The MathWorks Inc., MA, USA). The first five volumes from each run were discarded to allow for T1 equilibration effects. Preprocessing consisted of slice time correction, realignment and normalisation to the MNI template, and 8 mm FWHM smoothing. Additional filtering for seed based connectivity consisted of global drift removal using a 3rd order polynomial fit, with regression against: (i) motion parameters; (ii) mean white matter; (iii) mean grey matter; (iv) mean cerebrospinal fluid signal^58^. Finally, a phase-insensitive band-pass filter (pass band 0.01–0.08 Hz) was applied to reduce the effect of low frequency drift and high frequency physiological noise.

### General linear model analysis

Informed by our behavioural findings, neuroimaging analysis was focused on the naturalistic familiar and artificial familiar soundscape conditions. To explore differences in neural activity (inferred from haemodynamic changes in BOLD signal) within the DMN, random effects general linear model (GLM) analysis was conducted on the first level BOLD activation maps contrasting the artificial familiar (AF) against the naturalistic familiar (NF) conditions (AF > NF and NF > AF). An inclusive DMN mask was created from the acquired datasets, operationalized as the map of regions showing significant (*p*_(*FWE peak*)_ < 0.05) functional connectivity with the PCC seed in the no-sound control condition (see Method section: Seed based connectivity analysis). This map showed good agreement with previously described DMN regions^42^. Activation within the masked region was assessed for significant differences between the naturalistic and artificial conditions (AF > NF and NF > AF). Variations in activation associated with differences in the subjective ratings of pleasantness, intensity and distraction by the sounds themselves were removed from the model by including the difference in these terms as a nuisance covariate in the group (2^nd^ level) analysis. All 2^nd^ level maps were initially thresholded at *p*_(*unc. peak*)_ < 0.001, with a significance determined as a peak or cluster which survived FWE correction at *p* < 0.05.

### Seed based connectivity analysis

The DMN was identified via seed based functional neural connectivity using a PCC anatomical mask developed using whole-brain functional connectivity analysis of resting state networks in a large cohort of healthy control participants^18^. For each participant and condition, the BOLD signal timecourse was extracted from the region within the PCC mask, and averaged over all voxels. The seed signal was then entered as a regressor in the 1^st^ level model to identify regions with a significantly correlated BOLD timecourse over the duration of the run. Whole brain PCC functional connectivity was then contrasted between the artificial and naturalistic condition for each participant, with individual 1^st^ level contrasts taken forward to 2^nd^ level random effects analysis. As with the GLM analysis, variations in connectivity associated with differences in pleasantness, intensity and distraction by the sounds themselves were controlled for at the 2nd level by including the difference in these terms between conditions as nuisance regressors. This method of 1st level network identification and 2nd level comparison was also followed for exploratory analysis of the following networks using published anatomical seed regions^18^ (seed regions given in parenthesis):

1. Anterior salience (bilateral anterior insula);
2. Posterior salience (bilateral posterior insula);
3. Dorsal attention / visuospatial (right supramarginal gyrus);
4. Left executive control (left dorsolateral prefrontal cortex);
5. Right executive control (right dorsolateral prefrontal cortex).

All 2^nd^ level maps were initially thresholded at *p*_(*unc. peak*)_ < 0.001, with significance determined as a peak or cluster which survived FWE correction at *p* < 0.05. *Z*-scores of connectivity strength (Pearson’s correlation with the BOLD signal timecourse of the seed) were extracted from the peak of significant clusters (averaged over a 5 mm radius ROI) and entered into a separate three-way ANOVA for each network (naturalistic familiar, artificial familiar and no-soundscape) to assess the direction of connectivity differences. Appropriate corrections were performed where the assumption of sphericity was violated.

## Additional Information

**How to cite this article:** Gould van Praag, C. D. *et al*. Mind-wandering and alterations to default mode network connectivity when listening to naturalistic versus artificial sounds. *Sci. Rep.* **7**, 45273; doi: 10.1038/srep45273 (2017).

**Publisher's note:** Springer Nature remains neutral with regard to jurisdictional claims in published maps and institutional affiliations.
